# Multidrug-resistant ST11-KL64 hypervirulent *Klebsiella pneumoniae* with multiple *bla-* genes isolated from children's blood

**DOI:** 10.3389/fped.2024.1450201

**Published:** 2025-01-06

**Authors:** Rongmu Luo, Guannan Ma, Qian Yu, Zhengqin Tian, Qihang Man, Xiangrong Shu, Xuetong Liu, Yupeng Shi, Lei Zhang, Jingbo Wang

**Affiliations:** ^1^ Department of Hematology, Aerospace Center Hospital, Beijing, China; ^2^ Department of Hematology, China Aerospace Science & Industry Corporation 731 Hospital, Beijing, China; ^3^ Medical Research Center, Key Laboratory of Digital Technology in Medical Diagnostics of Zhejiang Province, Hangzhou, China

**Keywords:** hv-CRKP, polymyxin, tigecycline, carbapenem, BMT

## Abstract

**Introduction:**

Hypervirulent carbapenem-resistant Klebsiella pneumoniae (hv-CRKP) poses an increasing public health risk due to its high treatment difficulty and associated mortality, especially in bone marrow transplant (BMT) patients. The emergence of strains with multiple resistance mechanisms further complicates the management of these infections.

**Methods:**

We isolated and characterized a novel ST11-KL64 hv-CRKP strain from a pediatric bone marrow transplantation patient. Antimicrobial susceptibility testing was performed to determine resistance patterns. Comprehensive genomic analysis was conducted to identify plasmid types, virulence factors, and antimicrobial resistance genes, as well as potential resistance mechanisms associated with mutations and plasmid-mediated variants.

**Results:**

The isolated hv-CRKP strain exhibited multidrug resistance to carbapenem, tigecycline, and polymyxin. Genomic analysis revealed that the IncHI1B/repB plasmid carried virulence factors (*rmpA, ΔrmpA2, iucABCD, iutA*), while IncFII/IncR and IncFII plasmids harbored resistance genes [*bla*_*CTX-M-65*_*, bla*_*TEM-1B*_*, rmtB, bla*_*SHV-12*_*, bla*_*KPC-2*_*, qnrS1, bla*_*LAP-2*_*, sul2, dfrA14, tet(A), tet(R)*]. The coexistence of *bla*_*CTX-M-65*_*, bla*_*TEM-1B*_*, bla*_*SHV-12*_*, bla*_*LAP-2*_*,*and *bla*_*KPC-2*_ in one hv-CRKP strain is exceptionally rare. Additionally, the Tet(A)-S251A variant in the conjugative plasmid pTET-4 may confer tigecycline resistance. Mutations in MgrB, PhoPQ, and PmrABCDK were identified as potential contributors to increased polymyxin resistance. Interestingly, plasmid-encoded restriction-modification systems and Retron regions were identified, which could potentially confer phage resistance.

**Discussion:**

The combination of virulence and antimicrobial resistance factors in the ST11-KL64 hv-CRKP strain represents a significant challenge for treating immunocompromised pediatric patients. Particularly concerning is the resistance to polymyxin and tigecycline, which are often last-resort treatments for multidrug-resistant infections. The findings highlight the urgent need for effective surveillance, infection control measures, and novel therapeutic strategies to manage such hypervirulent and multidrug-resistant pathogens.

## Introduction

The prevalence of carbapenem-resistant *K. pneumonia* (CRKP) infection poses a significant and enduring obstacle to worldwide public health systems (1–3). The emergence of hypervirulent CRKP (hv-CRKP) strains has added another layer of complexity, as they combine high virulence with multidrug resistance, severely limiting treatment options and increasing mortality rates (4). ST11, a dominant CRKP clone in China, has emerged as a high-risk lineage due to its ability to acquire virulence plasmids (5). Zhou et al. reported that ST11-KL64 CRKP has gradually replaced ST11-KL47 and become the most prevalent and highly virulent CRKP clone in China since 2016 (6). This lineage poses a significant public health threat, particularly among immunocompromised individuals, due to its enhanced transmissibility, multidrug resistance, and hypervirulence (7). The propagation of CRKP among individuals with hematological malignancies presents a particularly worrisome challenge (8–11). Favorable conditions for the spread of CRKP infection include potential blood disorders, intensified chemotherapy, neutropenia, gastrointestinal mucositis, and prolonged hospitalization, all of which heighten the risk of bacteremia (8–10). The mortality rate associated with CRKP bacteremia in individuals with neutropenic hepatopathy has been reported to be approximately 60%, with a continued high prevalence expected, particularly among recipients of BMT (12, 13). Infections caused by ST11 hv-CRKP strains are further exacerbated by their rapid evolution of resistance under antibiotic pressure, with mechanisms involving ramR, mgrB, and pmrB mutations contributing to resistance to last-resort antibiotics like polymyxin and tigecycline (14). In addition, the emergence of hypervirulent *K. pneumoniae* (hvKP) has been observed at a higher rate (55.3%) in immunocompromised patients (11). However, there remains a paucity of research examining the impact of hv-CRKP on this high-risk population.

Currently, the therapeutic options available for hv-CRKP infection remain limited. Polymyxin and tigecycline are regarded as last-resort antibiotics in the management of CRKP-related infections (4, 15, 16). Regrettably, bacteria can develop resistance to polymyxin and tigecycline through alterations in mgrB, mutations in the two-component regulatory system (pmrABCDK and phoPQ), or disruption of regulatory genes that encode efflux pumps (e.g., ramR, ramA, and rarA) (17, 18). These resistance mechanisms further complicate the management of hv-CRKP infections and highlight the urgent need for novel therapeutic strategies.

Despite increasing attention to hv-CRKP infections, particularly in immunocompromised populations, there remains limited understanding of the genetic determinants underlying hypervirulence and multidrug resistance, especially in strains like ST11-KL64. Moreover, the concurrent presence of multiple resistance genes (e.g., bla_CTX-M-65_, bla_KPC-2_) and virulence factors (e.g., *rmpA*, *iucABCD*) in a single hv-CRKP strain raises questions about their roles in clinical outcomes and therapeutic challenges. In addition, transmission of hv-CRKP is closely linked to its presence in the gut, which makes it more widespread in community and healthcare Settings (19). To address these gaps, our study aims to comprehensively characterize the genomic features of the ST11-KL64 hv-CRKP strain CZC, with a focus on the mechanisms driving its hypervirulence and resistance to critical antibiotics. By elucidating these mechanisms, we hope to provide insights that could inform effective strategies for managing hv-CRKP infections in high-risk populations.

## Materials and methods

### Ethics approval and consent to participate

This study was reviewed and approved by the Aerospace Center Hospital (No. JHYLS-2022–122). Verbal informed consent was obtained from the subject in this study. This study informed verbal consent was obtained from participant.

### Medical history of infection

An 18-year-old male patient underwent allogeneic hematopoietic stem cell transplantation due to primary immunodeficiency disease. Soon afterwards, the patient was diagnosed with a bloodstream infection, and a CRKP isolate (named CZC) was isolated from the blood sample. Combination antibiotic treatment was used immediately, including meropenem, polymyxin, ceftazidime avibactam, vancomycin, and tigecycline. Unfortunately, the patient died from this infection.

### Isolation and identification of bacteria

*K. pneumoniae* CZC was isolated from the blood samples of this patient. Blood culture was done using a BACT/ALERT® 3D blood culture system(bioMérieux, France). Bacteria was confirmed using matrix-assisted laser desorption/ionization time-of-flight (MALDI-TOF) mass spectrometry(MS) (Vitek MS, bioMerieux, France). In addition, molecular genotyping (serotyping and multilocus sequence typing) and phenotype validation (antibiotic susceptibility assay and *Galleria mellonella* infection model) were examined.

### Phenotypic characterization

We employed the disk diffusion method in our testing. Antimicrobial susceptibility testing was conducted using GN335 cards from the Vitek 2 Compact system (bioMérieux, France), with broth microdilution and disk diffusion methods used to validate the results for certain antibiotics (20) Colistin was tested using the broth microdilution method, while meropenem, imipenem, and tigecycline were tested using the disk diffusion method and the results were interpreted according to the CLSI standards. The virulence phenotype was evaluated by the *G. mellonella* larvae infection model*.* NTUH-K2044 served as a hypervirulent control strain, while 13,190, verified by whole-genome sequencing to lack virulence genes, was used as a low-virulence control strain (21, 22). All experiments were performed with three replications. The statistical analysis was performed using GraphPad Prism software.

### Genomic DNA sequencing, assembly, correction, and annotation

Whole-genome sequencing was performed using the PromethION platform (Oxford Nanopore Technologies Inc., UK). This strain was resequenced using Illumina NovoSeq 6,000 sequencing platform (Illumina, CA, USA) to prevent or correct errors. The complete genome was generated by Unicycler v0.4.4 (23) and annotated using RAST (24). Information on the CZC strain has been submitted to the NCBI database with the project accession number PRJNA865496. The biosample number is SAMN30114398 and the accession numbers CP102390–CP102396.

### Bioinformatics analysis

The circular genome maps were drawn with Proksee (https://proksee.ca). The virulence genes were identified using the Pasteur Institute and VFanalyzer databases. The resistance genes were identified using ResFinder. The SNP-based phylogenetic tree was constructed using the Pathogenwatch phylogenetic tool (https://pathogen.watch), and the isolates were compared to twenty-nine strains of ST11-KL64 *K. pneumoniae* isolates and the genomes of the CZC strain, which is available in the Pathogenwatch database. The 1972 core genes library of *K. pneumoniae* were used in Pathogenwatch to generate pairwise single nucleotide polymorphism (SNP) distances between genomes, which are used to construct neighbor-joining trees (25). The information on resistance genes and isolated countries was derived from annotation information in Pathogenwatch. iToL was used for phylogenetic tree improvement (26). The incompatible plasmid groups were identified using PlasmidFinder. The type of plasmid was determined by comparison to the nucleotide database using BLAST.

## Results

### Patient history

The patient was diagnosed with splenomegaly at the age of 6, accompanied by recurrent fever and lymphadenopathy, which gradually progressed to pancytopenia. Genetic testing identified a mutation in the XIAP gene, confirming a diagnosis of X-linked lymphoproliferative disease. At 18 years old, the patient developed hemophagocytic syndrome, which showed poor response to chemotherapy. As a result, an allogeneic hematopoietic stem cell transplantation was performed, with the donor being a fully matched (10/10) unrelated hematopoietic stem cell donor. Two months post-transplant, the patient experienced sudden chills and rigor, with a temperature spike to 39°C. Blood pressure dropped to 80/50 mmHg, urine output became scant, and the heart rate increased to 130 beats per minute. Laboratory tests revealed IL-6 levels exceeding 4,000 ng/ml, PCT levels peaking at 59 ng/ml, and CRP levels above 150 ng/ml. Blood cultures confirmed the presence of *K. pneumoniae*. A chest x-ray performed the following day showed extensive pulmonary infiltrates in both lungs, accompanied by respiratory distress and type I respiratory failure. By the fourth day, the patient succumbed to multi-organ dysfunction, with sepsis and pulmonary ARDS identified as the primary causes of death.

### Resistance and virulence phenotypes of *K. pneumoniae* strains

**Figure 1:**
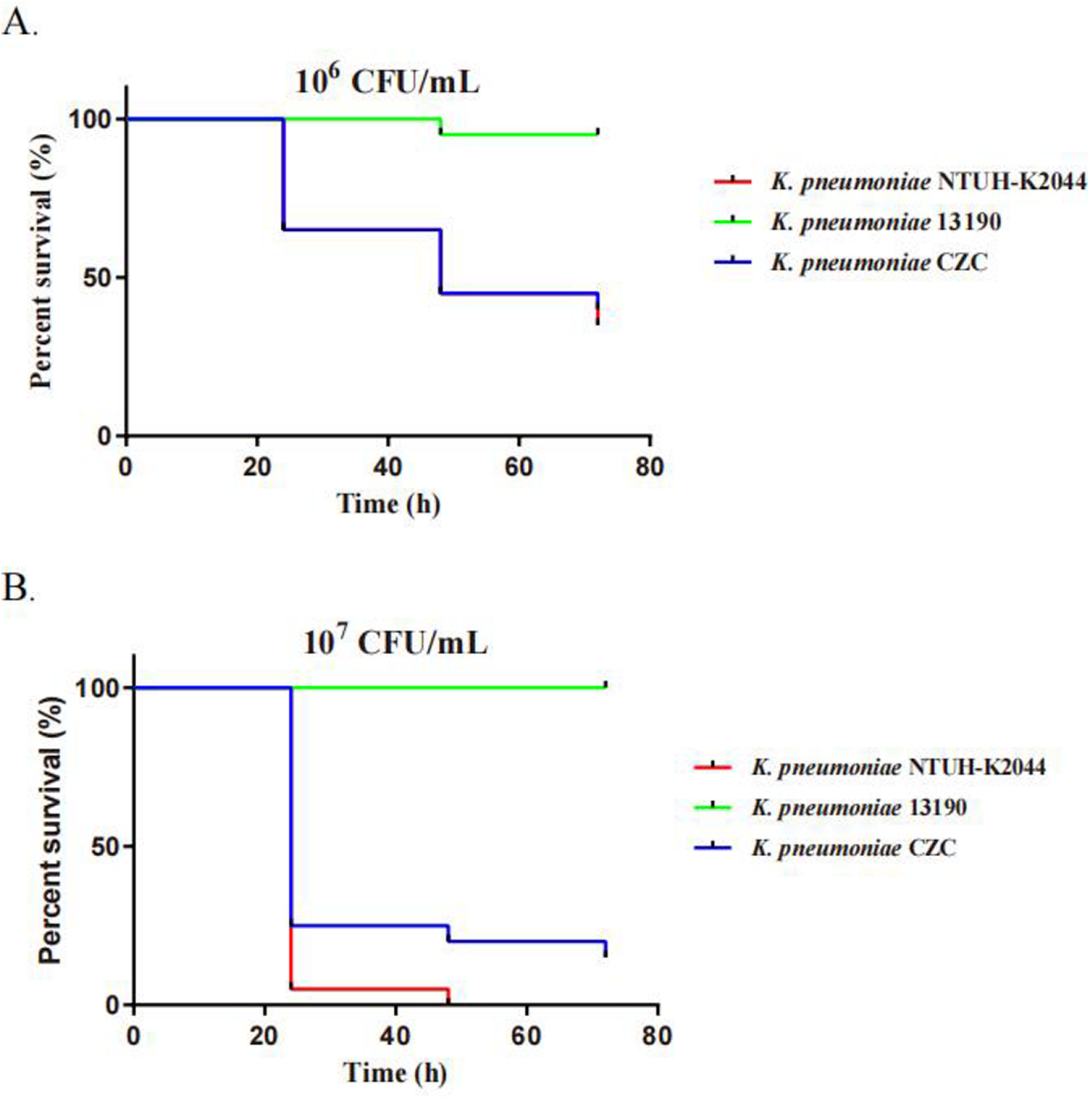
Virulence assays of three *K. pneumoniae* strains through a *Galleria mellonella* infection model. The virulence was determined by the survival rates of *G. mellonella* infected with the bacteria at two concentrations: **(A)** 1 × 10^6^ CFU/ml and **(B)** 1 × 10^7^ CFU/ml.

*K. pneumoniae* CZC showed resistance to a total of seventeen antibiotics in eleven categories. In particular, it was resistant to tigecycline, polymyxin, meropenem, imipenem and amikacin (Table 1). In addition, we performed testing using the disk diffusion method and broth microdilution method. The MIC value for colistin was ≥4.0 mg/L, for meropenem was ≥16.0 µg/ml, for imipenem was ≥16.0 µg/ml, and for tigecycline was ≥4 µg/ml, with an inhibition zone diameter of less than 14 mm. Additionally, for CZA tested by disk diffusion, the inhibition zone diameter was 22 mm, which is considered intermediate (I), indicating that the strain's resistance to CZA falls between susceptible and resistant. In addition, we used two control strains (hvKP strain NTUH-K2044 and CRKP strain 13,190) and *K. pneumoniae* CZC for virulence phenotype experiments. The *G. mellonella* larvae infected with *K. pneumoniae* CZC and NTUH-K2044 had significantly lower survival than those infected with CR-KP 13,190 (Figure 1). These results suggest that *K. pneumoniae* CZC is a hypervirulent strain.

**Table 1:** Antimicrobial resistances for *K. pneumoniae* CZC.

Drug	Abbreviation	MIC (mg/L)	Susceptibility
Minocycline	MNO	≥16.0	R
Doxycycline	DO	≥16.0	R
Tigecycline^a^^,^^b^	TGC	≥4.0	R
Ceftazidime/avibactam^b^	CZA	4	S
Colistin^a^	CS	≥4.0	R
Sulfamethoxazole	SXT	≥320.0	R
Ticarcillin/clavulnic acid	TCC	≥128.0	R
Piperacillin/Tazobactam	TZP	≥128.0	R
Aztreonam	ATM	≥64.0	R
Ciprofloxacin	CIP	≥4.0	R
Levofloxacin	LEV	≥8.0	R
Ceftazidim	CAZ	≥64.0	R
Cefoperazone/Sulbactam	SFP	≥64.0	R
Cefepime	FEP	≥32.0	R
Imipenem^a^	IPM	≥16.0	R
Meropenem^a^	MEM	≥16.0	R
Amikacin	AMK	≥64.0	R
Tobramycin	TOB	≥16.0	R

^a^ Broth microdilution method.

^b^ Disk diffusion method.

### Genomic characterization of the St11-Kl64 hv-CRKP CZC strain

To analyze the genetic characteristics of the strain, we performed Oxford Nanopore and Illumina genome sequencing and drew the complete genome map (Supplementary Figure S1), including a chromosome with 5,349,784 bp and six plasmids (pVir-1, pKPC-ESBL-2, pRM-3, pTET-4, pColRNAI-5, and p6) (Table 2).

**Table 2:** Genome information of *K. pneumoniae* CZC.

Sample Name	Genome size(bp)	Type	GC content (%)	Coding genes	Average gene sizes (bp)	Coding region(bp)	tRNAs	rRNAs
K.pneumoniae CZC	5,349,784	–	57.4	5,274	1,014.37	4,919,760	85	25
pVir-1	193,821	IncHI3_repHI3B_pNDM-MAR	50.4	235	824.77	175,199	0	0
pKPC-ESBL-2	135,425	IncFII/IncR	53.3	174	778.30	128,904	0	0
pRM-3	113,637	IncpKPHS1_repA_IncpKPHS1	49.1	116	979.63	99,973	1	0
pTET-4	84,876	IncFIIpKp_Goe_414-4	54.1	101	840.36	80,085	0	0
pColRNAI-5	11,934	ColRNAI	55.5	16	745.88	9,328	0	0
p6	5,596	–	51.1	12	466.33	3,132	0	0

According to the MLST and capsule type results, *K. pneumoniae* CZC belongs to the ST11/CG258 and KL64 capsule types.

**Figure 2:**
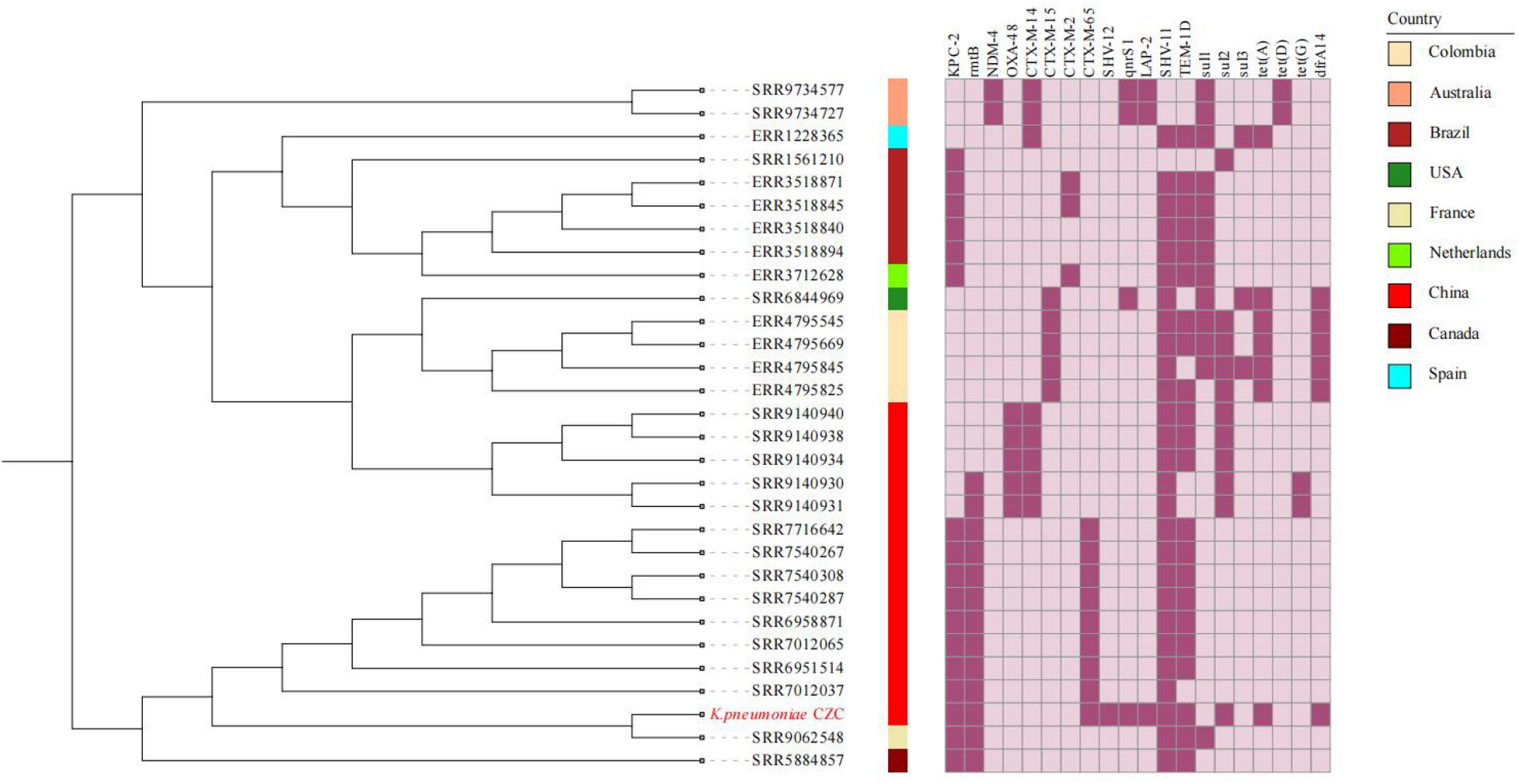
The phylogenetic tree and heatmap of AMR genes of ST11-KL64. The colored strips beside the phylogenetic tree indicate the countries. Strain CZC is highlighted in red.

Phylogenetic analysis indicates that the ST11-KL64 strains have developed a greater population clone (Figure 2). Most of the ST11-KL64 strains have drug resistance combinations *bla*_KPC-2_, *bla*_TEM-1B,_ and *bla*_SHV-11_ (Figure 2). The CZC strain coexisted with *bla*_CTX-M-65_, *bla*_TEM-1B_, *bla*_SHV-12_, *bla*_LAP-2_ and *bla*_KPC-2_, which was very rare in ST11-KL64 hv-CRKP strains.

### Virulence and drug resistance gene distribution

Antimicrobial resistance analysis showed that the strain contained resistance genes for *β*-lactamides *(bla*_TEM-1B_, *bla*_CTX-M-65,_ *bla*_SHV-11_, *bla*_LAP-2,_ and *bla*_SHV-1*2*_), carbapenems (*bla*_KPC-2_), fosfomycin (*fosA6*), tetracycline [*tet(A), tet(R)*], trimethoprim (*dfrA14*), sulfonamides (*sul2*), aminoglycosides (*rmtB*), efflux pumps (*CRP, lptD, kpnEFG*) and pmr phosphoethanolamine transferase (*eptB, arnT*) (Table 3). Meanwhile, pKPC-ESBL-2 (135,425 bp, IncFII/IncR) carried *bla*_CTX-M-65_, *bla*_TEM-1B_, *rmtB*, *bla*_SHV-12_, and *bla*_KPC-2_. pTET-4 (84,876 bp, IncFII) carried *qnrS1, bla_LAP-2_, sul2, dfrA14, tet(A), and tet(R)* (Supplementary Figure S2).

**Table 3:** AMR gene carriage profiles of *K. pneumoniae* CZC.

Orientation	AMR Gene	AMR Gene Family
Chromosome	*bla*SHV-11	SHV beta-lactamase
	*CRP*	RND antibiotic efflux pump
	*lptD*	ABC antibiotic efflux pump
	*kpnEFG*	MFS antibiotic efflux pump
	*eptB*	pmr phosphoethanolamine transferase
	*fosA6*	fosfomycin
	*arnT*	pmr phosphoethanolamine transferase
p2-CRKP-ESBL	*bla*CTX-M-65	CTX-M beta-lactamase
	*bla*TEM-1B	TEM beta-lactamase
	*rmtB*	16S rRNA methyltransferase (G1405)
	*bla*SHV-12	SHV beta-lactamase
	*bla*KPC-2	KPC beta-lactamase
p4-tet(A)	*qnrS1*	quinolone resistance protein (qnr)
	*bla*LAP-2	LAP beta-lactamase
	*sul2*	sulfonamide resistant sul
	*dfrA14*	trimethoprim resistant dihydrofolate reductase dfr
	*tet(A)*	MFS antibiotic efflux pump
	*tet(R)*	MFS antibiotic efflux pump

Based on the analysis of virulence genes, the CZC strain has 108 virulence genes, including capsular polysaccharide (*rmpA and ΔrmpA2*), type I fimbriae system (*fimABCDEFGHIK*), type 3 fimbriae system (*mrkABCDF*), aerobactin *(iucABCD, iutA, and ΔiroN*) and yersiniabactin *(fyuA, irp1, irp2, and ybtAEPQSTUX*). These virulence genes are recognized virulence factors and play important virulence roles. Meanwhile, pVir-1 (193,821 bp, IncHI1B/FIB), with *rmpA*, *ΔrmpA2*, *iucABCD*, *iutA,* and *ΔiroN,* is highly homologous to the virulence plasmid pLVPK (89% coverage, 99.43% identity) (Supplementary Figure S3). The IncFIB/IncHI1B type virulence plasmid could be transferred via conjugation to *Escherichia coli* and *K. pneumoniae* strains, leading to a sharp increase in the prevalence of hv-CRKP in clinical settings, which poses a great threat to human health (27).

### Comparative analysis of MDR-resistant regions

The MDR regions of *K. pneumoniae* CZC were mainly distributed on pKPC-ESBL-2 (IncFII/IncR) and pTET-4 (IncFII).

**Figure 3:**
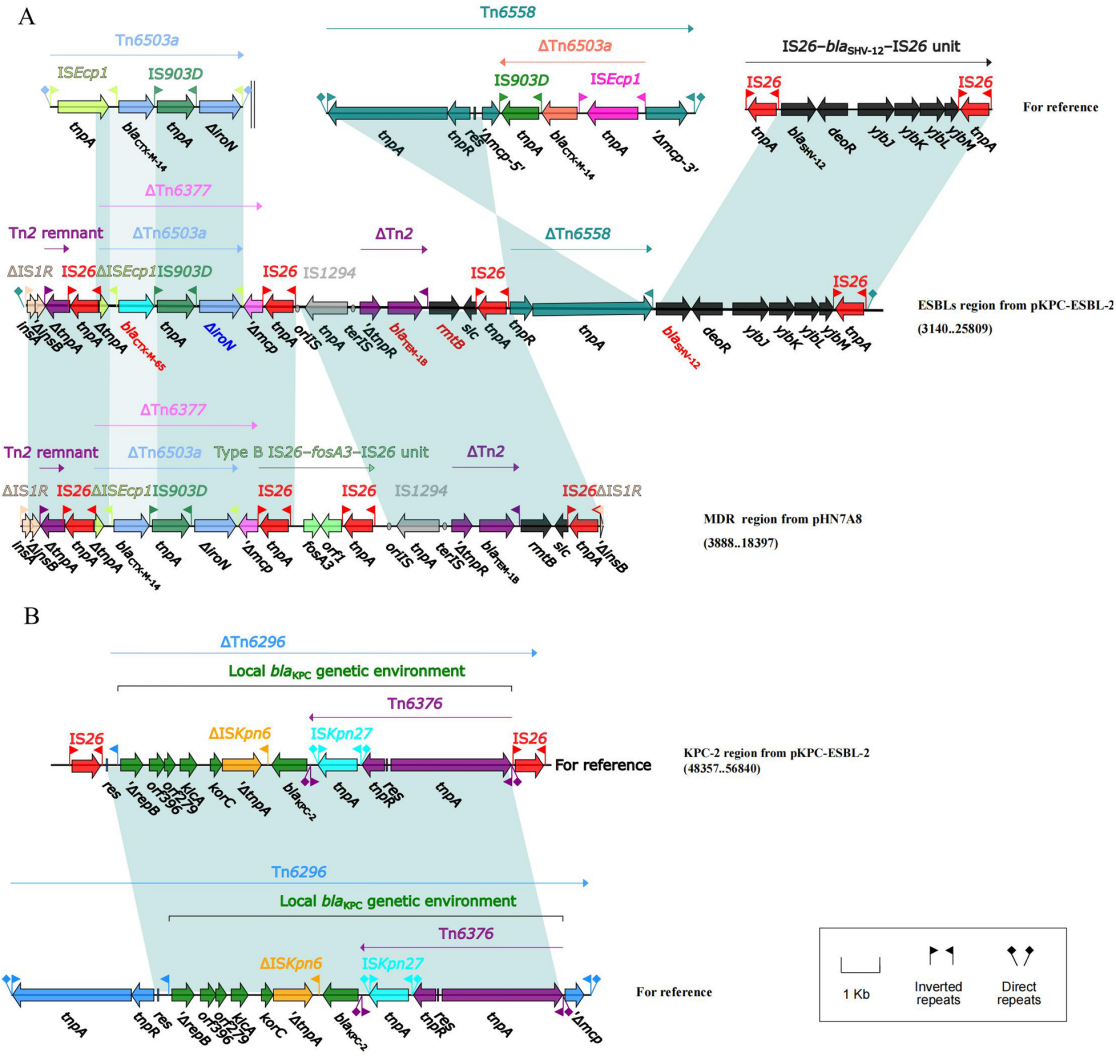
Lineal comparison of multidrug-resistant regions of pKPC-ESBL-2. Genes are denoted by arrows. Shading denotes the regions with high homology (≥95% nucleotide identity). **(A)** Comparison of the ESBLs region from pKPC-ESBL-2 and the MDR region from pIIN7A8. **(B)** Comparison of the KPC-2 region from pKPC-ESBL-2 with related genetic structures, illustrating the blaKPC-2 genetic environment.

pKPC-ESBL-2 was divided into two parts (Figure 3, Supplementary Figure S4). One part is the ESBL region containing *bla*_CTX-M-65_, *bla*_TEM-1B_, *rmtB*, and *bla*_SHV-12._ The *IS1R*-*IS26* element in the ends of the entire MDR region can mediate homologous recombination in the corresponding region (Figure 3A). The other part is the KPC-2 region, a truncated *Tn6296* flanked by an *IS26* insertion that can mediate the transfer of the *bla*_KPC-2_-resistant region (Figure 3B).

The MDR region on pTET-4 showed a high similarity to pKp_Goe_414-4 (CP018341.1, 95% coverage), containing the resistance genes *qnrS1, bla*_LAP-2_*, sul2, dfrA14, tet(A), and tet(R)* (Supplementary Figure S4).

### Point mutations are responsible for resistance to tigecycline and polymyxin

The consistency between the clinical and drug susceptibility results of the CZC strain supports insensitivity to polymyxin and tigecycline (Table 1). Genetic analysis revealed that tigecycline resistance was associated with the *tet(A)* gene, with the Tet(A)-S251A mutation identified on the conjugal plasmid pTET-4. This mutation correlated with increased MICs for tigecycline, tetracycline, and minocycline. Polymyxin resistance was linked to *mgrB*-M1V, *phoQ*-D150G, *pmrB*-R256G, *pmrC*-Q319R, *pmrD*-D80G, and *pmrK*-Q156H R157S mutations within the MgrB, PhoPQ, and PmrABCDK systems (Table 4). Additionally, the protein KpnEF in the CZC strain may play a role in capsule synthesis and resistance to polymyxin. The presence of a type I R-M system in pRM-3 and a predicted Retron region in pColRNAI-5 suggests potential antiphage defense mechanisms in the strain.

**Table 4:** Mgrb, PhoPQ and PmrABCDK mutations.

Name	Amino acid alterations							
	mgrB	phoP	phoQ	pmrA	pmrB	pmrC	pmrD	pmrK
CZC	M1V	WT	D150G	WT	R256G	Q319R	D80G	Q156H, R157S

Annotations of Amino acid alterations were based on the protein sequence of Klebsiella pneumoniae subsp. pneumoniae NTUH-K2044 (Accession no. NC_012731.1).

## Discussion

ST11 is a common clone group of CRKP in China, and it is also a high-risk clone group with the ability to obtain highly virulent plasmids (6). Zhou *et al*. reported that ST11-KL64 CRKP had gradually replaced ST11-KL47 and became the most prevalent and highly virulent CRKP clone in China since 2016 (28). In our study, a novel ST11-KL64 CZC strain was first isolated from the blood of pediatric BMT patient and was identified to have a high virulence and multidrug-resistant phenotype. The genome analysis indicates that hypervirulent *K. pneumoniae* with tigecycline, polymyxin and carbapenem resistance might result in final antibiotic treatment failure. The spread of these strains poses an enormous threat to public health.

The hypervirulent phenotype of CZC is mainly provided by the pLVPK-like virulence plasmid carrying the virulence genes *rmpA, ΔrmpA2, iucABCD*, and *iutA.* They play an important role in inhibiting phagocytosis of host cells, immune evasion, increasing bactericidal activity, and anti-phagocytosis (29), which increase hvKP strain survival in the host and spread from the bloodstream.

In terms of drug resistance, CZC shows resistance to tigecycline, polymyxin, meropenem, and imipenem (Table 1). The CRKP resistance gene *bla*_KPC-2_ is located on the MDR plasmid pKPC-ESBL-2. It has an IncFII (pHN7A8)/IncR backbone containing two MDR regions (ESBLs and KPC-2), which is different from the structure of pHN7A8. The ESBL resistance region contain an IS26-*bla*_SHV-12_-IS26 unit and the *bla*_KPC-2_ resistant unit. In China, *bla*_KPC-2_ is most commonly found in an environment with an *IS26*-*Tn6296* compound transposon (30). The type of IncFII/IncR plasmid plays an important role in the spread of CRKP, carrying various resistance genes effectively (31). The IncFII/IncR-type *bla_KPC_*_-2_-bearing plasmid could be transferred via conjugation to Enterobacteriaceae strains alone as well as together with the IncFIB/IncHI1B-type virulence plasmid (32). Another resistance plasmid, pTET-4, has an IncFIIpKp_Goe_414-4-type backbone with *qnrS1*, *bla*_LAP-2_, *sul2, dfrA14, tet (A), and tet(R).* They were located in an MDR region (*ΔTn6591-ΔTn1721* region and truncated *IS26*–*bla*_LAP-2_–*qnrS1*–*IS26* unit). The results showed that *IS26* as well as transposons and integrons are essential for increasing multidrug resistance in *K. pneumoniae*.

Our analysis identified the Tet(A)-S251A mutation on the conjugal plasmid pTET-4, which has been previously shown to confer high-level tigecycline resistance in CRKP strains and *E. coli* ATCC 25,922, alongside increased MICs for tetracycline and minocycline (32, 33). Furthermore, the synergy between the *tet (A)* variant and the RND-type efflux transporters contributes to tigecycline resistance in *A. baumannii* (34), and this may also work in *K. pneumoniae*. Furthermore, sporadically reported mutations of genes (*pmrBCDK*, *phoQ*, and *mgrB*) even simultaneously occurred to acquire polymyxin resistance in our CZC isolate. These include mgrB-M1V, phoQ-D150G, pmrB-R256G, pmrC-Q319R, pmrD-D80G, and pmrK-Q156H R157S mutations within the MgrB, PhoPQ, and PmrABCDK systems, consistent with their known roles in elevating polymyxin MICs (21). Additionally, the KpnEF protein may contribute to capsule synthesis regulation and resistance mechanisms, as reported in *E. coli* (35).

With the complication of bacterial resistance, there is an urgent need for adequate antimicrobial agents to treat hv-CRKP infections. Tigecycline is regarded as a last resort treatment for CRKP infections and is widely used in clinical practice (36, 37). Therefore, the emergence of tigecycline and polymyxin resistance in hv-CRKP infection makes clinical treatment difficult (4) due to amino acid mutations in common targets. In addition to mutations in resistance genes, the presence of an antibiotic efflux pump in this strain may also have implications for its increased antimicrobial drug resistance (33). The involvement of Tet(A)-S251A and MgrB-M1V*,* and the synergy of the RND-type efflux transporters in the mechanisms of tigecycline resistance are worthy of further study. In addition, the Tet (A) S251A variant is located in the conjugative plasmid pTET-4, which provides a genetic basis for cotransfer with plasmids.

Furthermore, a type I R-M system and a predicted Retron region contained in the plasmid of CZC provide antiphage defense to the strain (38). In conclusion, the ST11-KL64 hv-CRKP CZC strain showed worrying results regarding antibiotic resistance and virulence. Our results suggest the potential of this strain to become a novel significant superbug and a threat to public health. In particular, it presents a deadly threat to immunocompromised patients.

Our study builds on existing knowledge of ST11-KL64 CRKP strains, which have been widely recognized as high-risk clones with significant virulence and resistance potential (28). Previous works have documented the gradual replacement of ST11-KL47 by ST11-KL64 as the dominant CRKP clone in China, driven by its enhanced virulence and transmissibility (28). Similarly, the hypervirulent phenotype in hv-CRKP has been previously attributed to pLVPK-like plasmids carrying virulence genes like rmpA and iucABCD (31). However, our study provides unique insights by identifying a novel ST11-KL64 hv-CRKP strain (CZC) from a pediatric BMT patient. Unlike previous reports, we demonstrated the coexistence of hypervirulence and resistance to carbapenems, tigecycline, and polymyxin within this strain. Specifically, we uncovered the genetic mechanisms of tigecycline resistance through the Tet(A)-S251A mutation and its synergy with RND-type efflux transporters, a phenomenon rarely studied in K. pneumoniae (35). Furthermore, the identification of mgrB and phoQ mutations in polymyxin resistance aligns with previous findings (21) but also expands the understanding by highlighting their co-occurrence with additional resistance determinants within the CZC strain.

## Conclusions

Based on a drug-susceptibility assay and a *G. mellonella* larvae infection model, we first confirmed the coexistence of hypervirulence and polymyxin, tigecycline, and carbapenem resistance in the ST11-KL64 hv-CRKP CZC strain. Further, we confirmed the genetic basis of these phenotypes through complete genome sequencing. Notably, resistance to polymyxin and tigecycline is a fatal threat to immunocompromised pediatric patients, especially those with BMT.

## Data Availability

The datasets presented in this study can be found in online repositories. The names of the repository/repositories and accession number(s) can be found in the article/Materials and methods.

## Supplementary material

Supplementary Figure S1 Circular representation of the K. pneumoniae CZC genome. From outside to inside, rings 1 and 2 are annotated coding genes and noncoding sequences (including tRNA, rRNA and tmRNA). The GC skew pattern is represented by the innermost ring, with purple indicating negative values and green indicating positive values.

Supplementary Figure S2 Lineal comparison of multidrug-resistant regions of pTET-4. Genes are denoted by arrows. Shading denotes the regions with high homology (≥95% nucleotide identity).

Supplementary Figure S3 pVir-1 Plasmid Schematic map and comparison.

Supplementary Figure S4 pKPC-ESBL-2 Plasmid Schematic map and comparison.

Supplementary Figure S5 pTET-4 Plasmid Schematic map and comparison.

Supplementary Figure S6 pColRNAI-5 Plasmid Schematic map and comparison.

## References

[1] Lutgring JD. Carbapenem-resistant Enterobacteriaceae: an emerging bacterial threat. Semin Diagn Pathol. (2019) 36:182–6. 10.1053/j.semdp.2019.04.011 31056277

[2] Wang Q Wang X Wang J Ouyang P Jin C Wang R Phenotypic and genotypic characterization of carbapenem-resistant Enterobacteriaceae: data from a longitudinal large-scale CRE study in China (2012–2016). Clin Infect Dis. (2018) 67(Suppl 2):S196–205. 10.1093/cid/ciy660 30423057

[3] Yang X Sun Q Li J Jiang Y Li Y Lin J Molecular epidemiology of carbapenem-resistant hypervirulent *Klebsiella pneumoniae* in China. Emerg Microbes Infect. (2022) 11:841–9. 10.1080/22221751.2022.2049458 35236251 PMC8942559

[4] Jin X Chen Q Shen F Jiang Y Wu X Hua X Resistance evolution of hypervirulent carbapenem-resistant *Klebsiella pneumoniae* ST11 during treatment with tigecycline and polymyxin. Emerging Microbes Infect. (2021) 10:1129–36. 10.1080/22221751.2021.1937327 34074225 PMC8205050

[5] Huang J Yi M Yuan Y Xia P Yang B Liao J Emergence of a fatal ST11-KL64 tigecycline-resistant hypervirulent *Klebsiella pneumoniae* clone cocarrying bla(NDM) and bla(KPC) in plasmids. Microbiol Spectr. (2022) 10:e0253922. 10.1128/spectrum.02539-22 36205391 PMC9769963

[6] Liao W Liu Y Zhang W. Virulence evolution, molecular mechanisms of resistance and prevalence of ST11 carbapenem-resistant *Klebsiella pneumoniae* in China: a review over the last 10 years. J Glob Antimicrob Resist. (2020) 23:174–80. 10.1016/j.jgar.2020.09.004 32971292

[7] Song S Zhao S Wang W Jiang F Sun J Ma P Characterization of ST11 and ST15 carbapenem-resistant hypervirulent *Klebsiella pneumoniae* from patients with ventilator-associated pneumonia. Infect Drug Resist. (2023) 16:6017–28. 10.2147/IDR.S426901 37705511 PMC10496924

[8] Perez F Adachi J Bonomo RA. Antibiotic-resistant gram-negative bacterial infections in patients with cancer. Clin Infect Dis. (2014) 59(Suppl 5):S335–339. 10.1093/cid/ciu612 25352627 PMC4303050

[9] Pagano L Caira M Trecarichi EM Spanu T Di Blasi R Sica S Carbapenemase-producing *Klebsiella pneumoniae* and hematologic malignancies. Emerg Infect Dis. (2014) 20:1235–6. 10.3201/eid2007.130094 24960464 PMC4073839

[10] Freire MP Pierrotti LC Filho HHC Ibrahim KY Magri ASGK Bonazzi PR Infection with *Klebsiella pneumoniae* carbapenemase (KPC)-producing *Klebsiella pneumoniae* in cancer patients. Eur J Clin Microbiol Infect Dis. (2015) 34:277–86. 10.1007/s10096-014-2233-5 25169967

[11] Khrulnova S Fedorova A Frolova I Tandilova K Likold E Klyasova G. Distribution of virulence genes and capsule types in *Klebsiella pneumoniae* among bloodstream isolates from patients with hematological malignancies. Diagn Microbiol Infect Dis. (2022) 104:115744. 10.1016/j.diagmicrobio.2022.115744 35872039

[12] Girmenia C Rossolini GM Piciocchi A Bertaina A Pisapia G Pastore D Infections by carbapenem-resistant *Klebsiella pneumoniae* in SCT recipients: a nationwide retrospective survey from Italy. Bone Marrow Transplant. (2015) 50:282–8. 10.1038/bmt.2014.231 25310302

[13] Satlin MJ Calfee DP Chen L Fauntleroy KA Wilson SJ Jenkins SG Emergence of carbapenem-resistant Enterobacteriaceae as causes of bloodstream infections in patients with hematologic malignancies. Leuk Lymphoma. (2013) 54:799–806. 10.3109/10428194.2012.723210 22916826

[14] Zhou C Zhang H Xu M Liu Y Yuan B Lin Y Within-Host resistance and virulence evolution of a hypervirulent carbapenem-resistant *Klebsiella pneumoniae* ST11 under antibiotic pressure. Infect Drug Resist. (2023) 16:7255–70. 10.2147/IDR.S436128 38023413 PMC10658960

[15] Sheu CC Chang YT Lin SY Chen YH Hsueh PR. Infections caused by carbapenem-resistant Enterobacteriaceae: an update on therapeutic options. Front Microbiol. (2019) 10:80. 10.3389/fmicb.2019.00080 30761114 PMC6363665

[16] Tsuji BT Pogue JM Zavascki AP Paul M Daikos GL Forrest A International consensus guidelines for the optimal use of the polymyxins: endorsed by the American college of clinical pharmacy (ACCP), European society of clinical microbiology and infectious diseases (ESCMID), Infectious Diseases Society of America (IDSA), international society for anti-infective pharmacology (ISAP), society of critical care medicine (SCCM), and society of infectious diseases pharmacists (SIDP). Pharmacotherapy. (2019) 39:10–39. 10.1002/phar.2209 30710469 PMC7437259

[17] Liu S Ding Y Xu Y Li Z Zeng Z Liu J. An outbreak of extensively drug-resistant and hypervirulent *Klebsiella pneumoniae* in an intensive care unit of a teaching hospital in southwest China. Front Cell Infect Microbiol. (2022) 12:979219. 10.3389/fcimb.2022.979219 36176583 PMC9513609

[18] Galani I Karaiskos I Giamarellou H. Multidrug-resistant *Klebsiella pneumoniae*: mechanisms of resistance including updated data for novel beta-lactam-beta-lactamase inhibitor combinations. Expert Rev Anti Infect Ther. (2021) 19:1457–68. 10.1080/14787210.2021.1924674 33945387

[19] Han X Yao J He J Liu H Jiang Y Zhao D Clinical and laboratory insights into the threat of hypervirulent *Klebsiella pneumoniae*. Int J Antimicrob Agents. (2024) 64:107275. 10.1016/j.ijantimicag.2024.107275 39002700

[20] Sharif N Ahmed SN Khandaker S Monifa NH Abusharha A Vargas DLR Multidrug resistance pattern and molecular epidemiology of pathogens among children with diarrhea in Bangladesh, 2019–2021. Sci Rep 2023:13:13975. 10.1038/s41598-023-41174-6 37634040 PMC10460387

[21] Liu X Wu Y Zhu Y Jia P Li X Jia X Emergence of colistin-resistant hypervirulent *Klebsiella pneumoniae* (CoR-HvKp) in China. Emerging Microbes Infect. (2022) 11:648–61. 10.1080/22221751.2022.2036078 35086435 PMC8896207

[22] Shen D Ma G Li C Jia X Qin C Yang T Emergence of a multidrug-resistant hypervirulent *Klebsiella pneumoniae* sequence type 23 strain with a rare bla(CTX-M-24)-harboring virulence plasmid. Antimicrob Agents Chemother. (2019) 63:e02273-18. 10.1128/AAC.02273-18 30602516 PMC6395898

[23] Wick RR Judd LM Gorrie CL Holt KE. Unicycler: resolving bacterial genome assemblies from short and long sequencing reads. PLoS Comput Biol. (2017) 13:e1005595. 10.1371/journal.pcbi.1005595 28594827 PMC5481147

[24] Aziz RK Bartels D Best AA DeJongh M Disz T Edwards RA The RAST server: rapid annotations using subsystems technology. BMC Genomics. (2008) 9:75. 10.1186/1471-2164-9-75 18261238 PMC2265698

[25] Argimon S David S Underwood A Abrudan M Wheeler NE Kekre M Rapid genomic characterization and global surveillance of Klebsiella using pathogenwatch. Clinical Infectious Diseases: an Official Publication of the Infectious Diseases Society of America. (2021) 73:S325–35. 10.1093/cid/ciab784 34850838 PMC8634497

[26] Letunic I Bork P. Interactive tree of life (iTOL) v5: an online tool for phylogenetic tree display and annotation. Nucleic Acids Res. (2021) 49:W293–6. 10.1093/nar/gkab301 33885785 PMC8265157

[27] Yang X Dong N Liu X Yang C Ye L Chan EW-C Co-conjugation of virulence plasmid and KPC plasmid in a clinical *Klebsiella pneumoniae* strain. Front Microbiol. (2021) 12:739461. 10.3389/fmicb.2021.739461 34819921 PMC8606748

[28] Zhou K Xiao T David S Wang Q Zhou Y Guo L Novel subclone of carbapenem-resistant *Klebsiella pneumoniae* sequence type 11 with enhanced virulence and transmissibility, China. Emerg Infect Dis. (2020) 26:289–97. 10.3201/eid2602.190594 31961299 PMC6986851

[29] Russo TA Marr CM. Hypervirulent *Klebsiella pneumoniae*. Clin Microbiol Rev. (2019) 32:e00001-19. 10.1128/CMR.00001-19 31092506 PMC6589860

[30] Wang L Fang H Feng J Yin Z Xie X Zhu X Complete sequences of KPC-2-encoding plasmid p628-KPC and CTX-M-55-encoding p628-CTXM coexisted in *Klebsiella pneumoniae*. Front Microbiol. (2015) 6:838. 10.3389/fmicb.2015.00838 26347725 PMC4541600

[31] Li C Jiang X Yang T Ju Y Yin Z Yue L Genomic epidemiology of carbapenemase-producing *Klebsiella pneumoniae* in China. Genomics Proteomics Bioinformatics. (2022) 20:1154–1167. 10.1016/j.gpb.2022.02.005 35307590 PMC10225488

[32] Du X He F Shi Q Zhao F Xu J Fu Y The rapid emergence of tigecycline resistance in bla(KPC-2) harboring *Klebsiella pneumoniae*, as mediated *in vivo* by mutation in tetA during tigecycline treatment. Front Microbiol. (2018) 9:648. 10.3389/fmicb.2018.00648 29675006 PMC5895649

[33] Chiu S-K Huang L-Y Chen H Tsai Y-K Liou C-H Lin J-C Roles of ramR and tet(A) mutations in conferring tigecycline resistance in carbapenem-resistant *Klebsiella pneumoniae* clinical isolates. Antimicrob Agents Chemother. (2017) 61:e00391-17. 10.1128/AAC.00391-17 28533243 PMC5527587

[34] Foong WE Wilhelm J Tam HK Pos KM. Tigecycline efflux in Acinetobacter baumannii is mediated by TetA in synergy with RND-type efflux transporters. J Antimicrob Chemother. (2020) 75:1135–9. 10.1093/jac/dkaa015 32049277

[35] Elizabeth R Baishya S Kalita B Wangkheimayum J Choudhury MD Chanda DD Colistin exposure enhances expression of eptB in colistin-resistant Escherichia coli co-harboring mcr-1. Sci Rep. (2022) 12:1348. 10.1038/s41598-022-05435-0 35079093 PMC8789769

[36] Poirel L Jayol A Nordmann P. Polymyxins: antibacterial activity, susceptibility testing, and resistance mechanisms encoded by plasmids or chromosomes. Clin Microbiol Rev. (2017) 30:557–96. 10.1128/CMR.00064-16 28275006 PMC5355641

[37] Roy S Datta S Viswanathan R Singh AK Basu S. Tigecycline susceptibility in *Klebsiella pneumoniae* and Escherichia coli causing neonatal septicaemia (2007–10) and role of an efflux pump in tigecycline non-susceptibility. J Antimicrob Chemother. (2013) 68:1036–42. 10.1093/jac/dks535 23335112

[38] Millman A Bernheim A Stokar-Avihail A Fedorenko T Voichek M Leavitt A Bacterial retrons function in anti-phage defense. Cell. (2020) 183:1551–61.12. 10.1016/j.cell.2020.09.065 33157039

