# Supplementary figures and images for: Multidrug-resistant ST11-KL64 hypervirulent Klebsiella pneumoniae with multiple bla- genes isolated from children's blood
Source: Front Pediatr. 2025 Jan 6;12:1450201. doi: 10.3389/fped.2024.1450201 (PMC11743731; doi:10.3389/fped.2024.1450201)

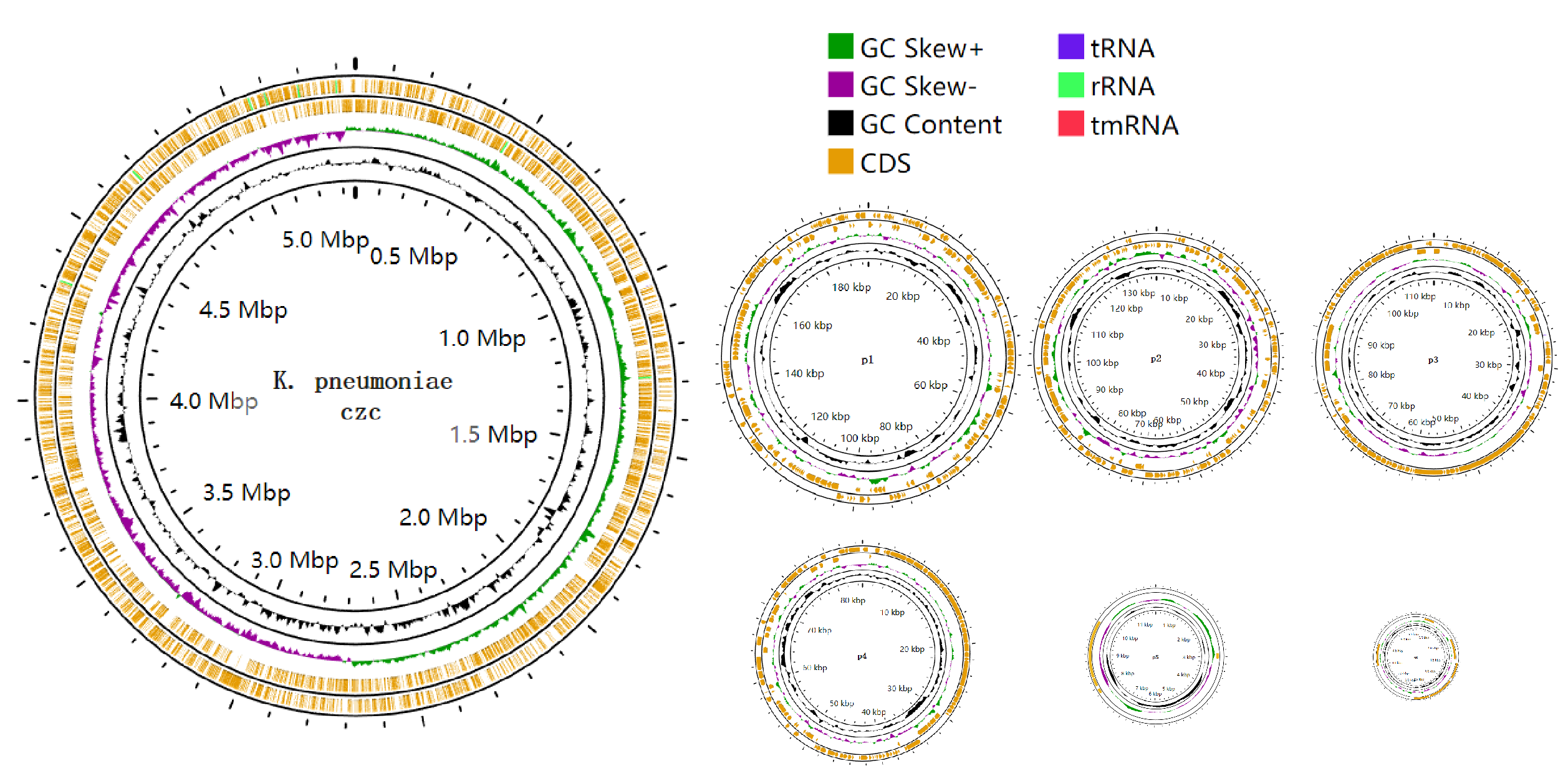

Supplement: Supplementary Figure S1 — Circular representation of the K. pneumoniae CZC genome. From outside to inside, rings 1 and 2 are annotated coding genes and noncoding sequences (including tRNA, rRNA and tmRNA). The GC skew pattern is represented by the innermost ring, with purple indicating negative values and green indicating positive values. [file Image1.tiff]

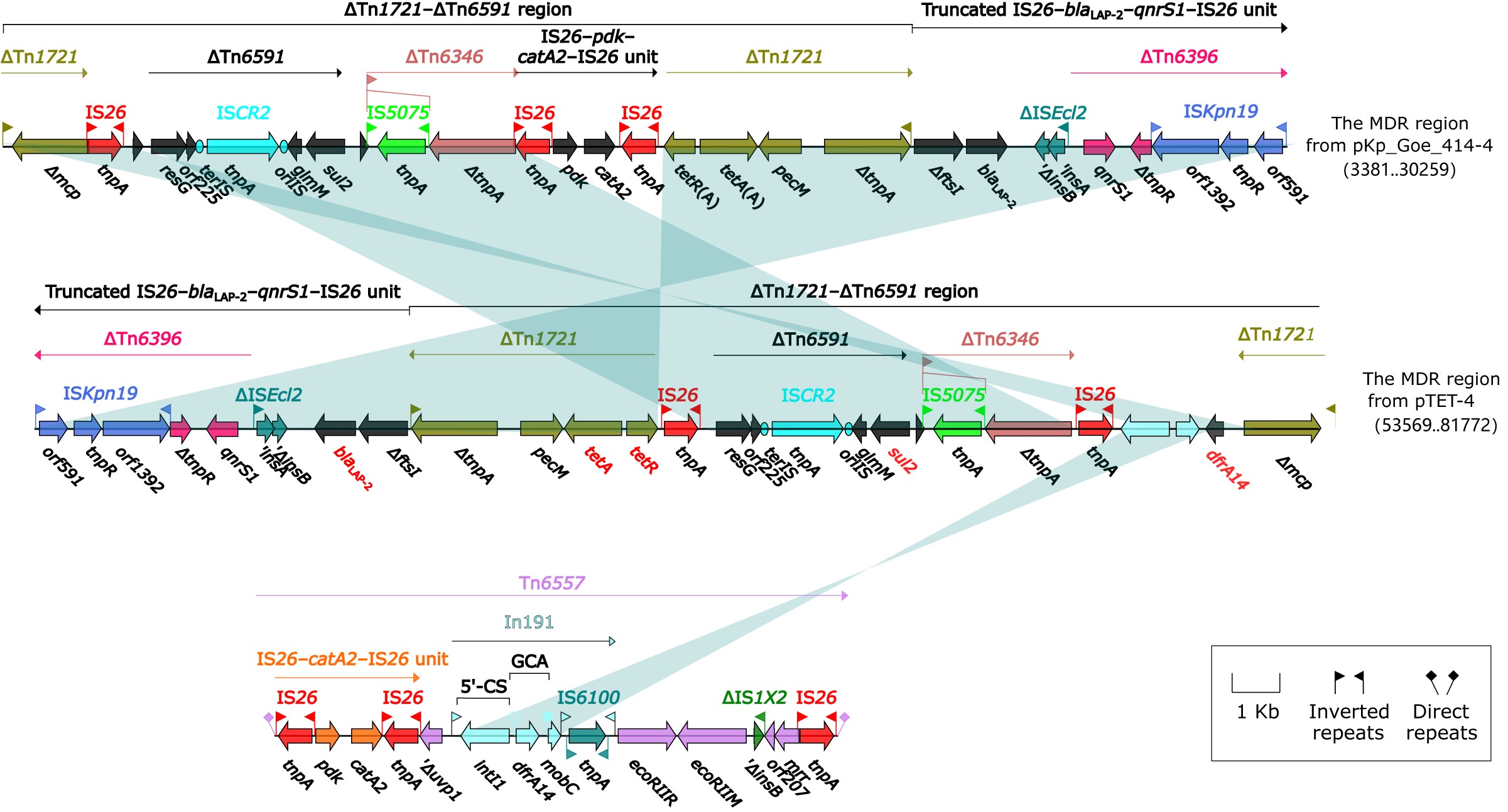

Supplement: Supplementary Figure S2 — Lineal comparison of multidrug-resistant regions of pTET-4. Genes are denoted by arrows. Shading denotes the regions with high homology (≥95% nucleotide identity). [file Image2.tiff]

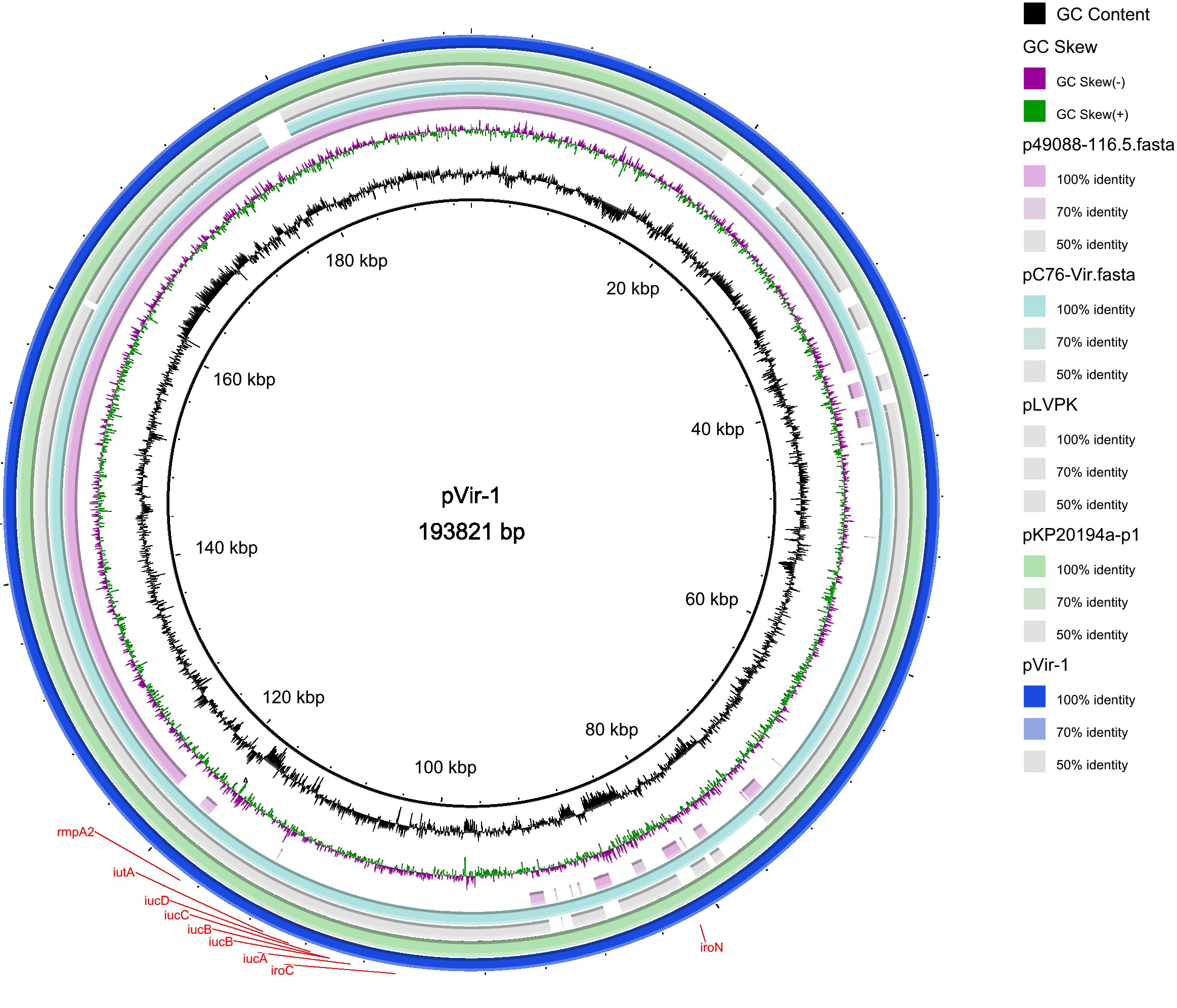

Supplement: Supplementary Figure S3 — pVir-1 Plasmid Schematic map and comparison. [file Image3.tiff]

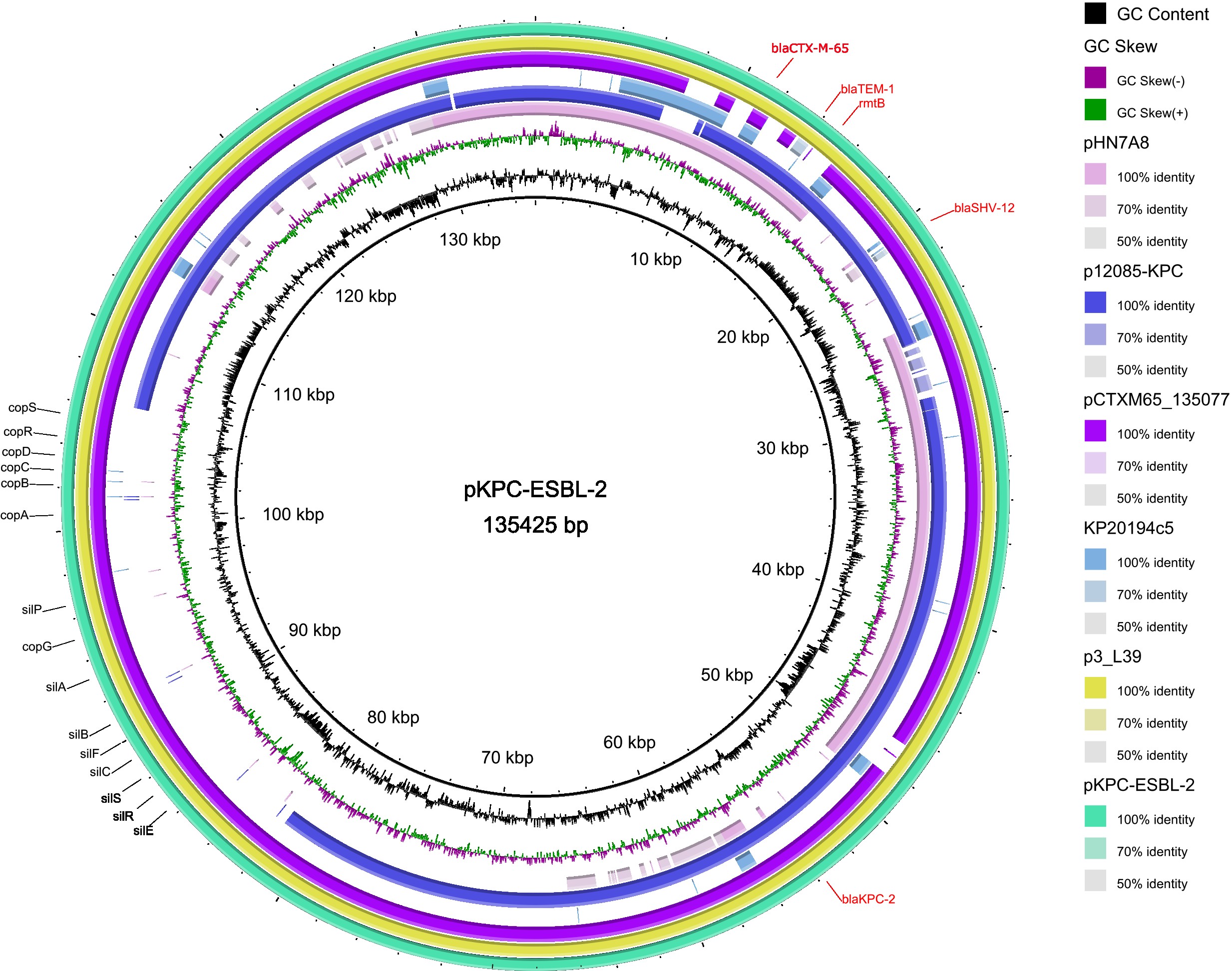

Supplement: Supplementary Figure S4 — pKPC-ESBL-2 Plasmid Schematic map and comparison. [file Image4.tiff]

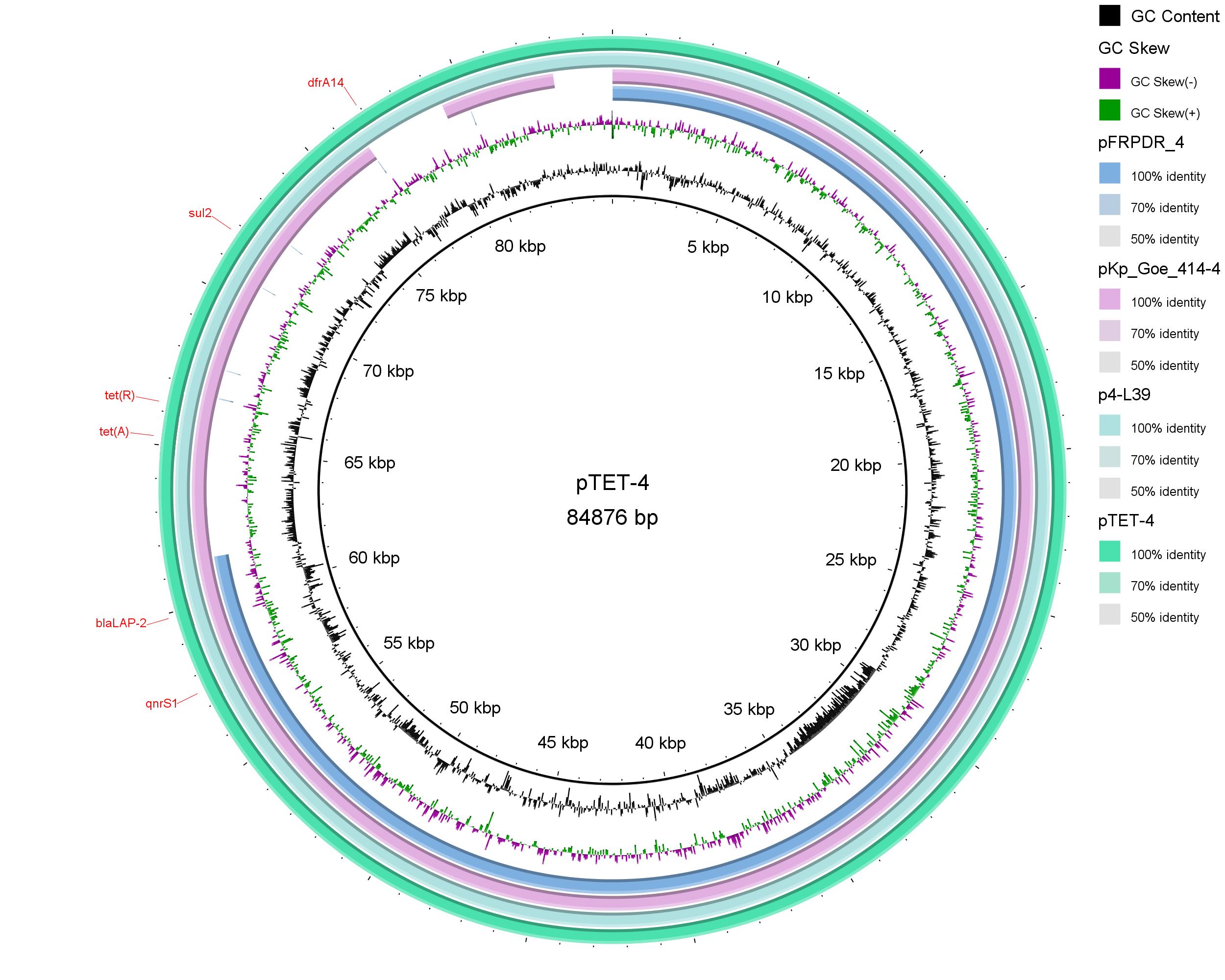

Supplement: Supplementary Figure S5 — pTET-4 Plasmid Schematic map and comparison. [file Image5.tiff]

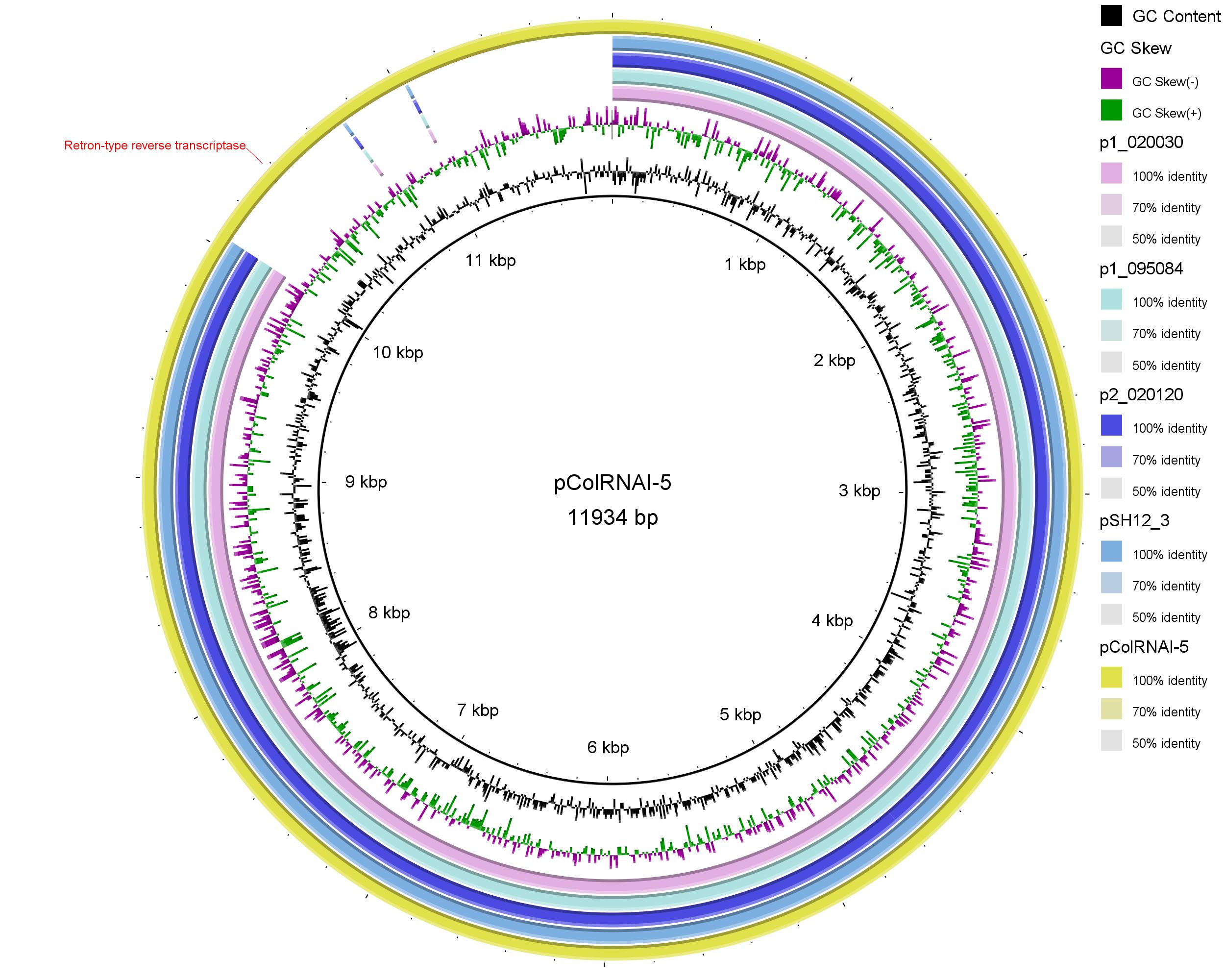

Supplement: Supplementary Figure S6 — pColRNAI-5 Plasmid Schematic map and comparison. [file Image6.tiff]
